# Human Allogeneic Bone Marrow-Derived Mesenchymal Stem Cell Therapy for Cerebellar Ataxia: A Case Report

**DOI:** 10.3390/medicina57040334

**Published:** 2021-04-01

**Authors:** Pan-Woo Ko, Sangmin Park, Kyunghun Kang, Yong-Hyun Lim, Sang Ryong Kim, Kyoungho Suk, Kyung Suk Kim, Ho-Won Lee

**Affiliations:** 1 Department of Neurology, Daegu Health College Hospital, Daegu 41422, Korea; Panwoo.ko@gmail.com; 2 Department of Neurology, Kyungpook National University Chilgok Hospital, School of Medicine, Kyungpook National University, Daegu 41404, Korea; eunsujeo@gmail.com (S.P.); knu.khkang@gmail.com (K.K.); 3 Center of Self-Organizing Software-Platform, Kyungpook National University, Daegu 41405, Korea; janggilsan21@gmail.com; 4 School of Life Sciences, BK21 Plus KNU Creative BioResearch Group, Kyungpook National University, Daegu 41566, Korea; srk75@knu.ac.kr; 5 Department of Pharmacology and Biomedical Science, School of Medicine, Kyungpook National University, Daegu 41944, Korea; ksuk@knu.ac.kr; 6 Bioengineering Institute, Corestem Inc., Seoul 13486, Korea; kskim@corestem.com

**Keywords:** mesenchymal stem cell therapy, allogeneic, cerebellar ataxia, sporadic adult-onset ataxia

## Abstract

To date, there is no curable treatment option for non-hereditary degenerative cerebellar ataxia. Here we report the case of a patient with sporadic adult-onset ataxia (SAOA) who underwent allogeneic bone marrow-derived mesenchymal stem cell (MSC) therapy via the intrathecal route. A 60-year-old male patient visited our clinic complaining of progressive gait disturbance that commenced two years ago. Upon neurologic examination, the patient exhibited limb dysmetria and gait ataxia. Brain magnetic resonance imaging (MRI) revealed cerebellar atrophy whereas the autonomic function test was normal. The patient was diagnosed with SAOA. The medications that were initially prescribed had no significant effects on the course of this disease and the symptoms deteriorated progressively. At the age of 64, the patient was treated with allogeneic bone marrow-derived MSC therapy. The subsequent K-SARA (Korean version of the Scale for the Assessment and Rating of Ataxia) scores demonstrated a distinct improvement up until 10 months post-administration. No adverse events were reported. The improved post-treatment K-SARA scores may suggest that the MSC therapy can have a neuroprotective effect and that stem cell therapy may serve as a potential therapeutic option for degenerative cerebellar ataxia.

## 1. Introduction

Cerebellar ataxia is an impairment of motor coordination and balance associated with the dysfunction of the cerebellum and its afferent and efferent pathways [1]. To date, there is no curable option for non-hereditary degenerative cerebellar ataxia. The development of disease-modifying agents has been attempted; however, the actual effect has not been achieved [2]. Mesenchymal stem cells (MSCs) are candidates for agents related to neuroprotection, the regeneration of neurons and anti-inflammatory reactions [3,4]. Herein, we report a case of a sporadic adult-onset ataxia (SAOA) patient that was treated with allogeneic bone marrow-derived MSC therapy via the intrathecal route.

## 2. Case Presentation

### 2.1. Methodology

The MSC (CS20BR08) used in this study was developed at Corestem’s Good Manufacturing Practice (GMP) verified facility and originated from haplotype matched bone marrow from a donor. Approximately 90 mL of bone marrow was donated by means of bone marrow aspiration and the whole bone marrow was separated as mononuclear cells (MNCs) using the Ficoll isolating method (Ficoll-PaqueTM Premium, GE Healthcare Bio-Sciences AB, Uppsala, Sweden). The separated MNCs were seeded on a T175 flask (Thermo Scientific Nunc, Roskilde, Denmark) and we then only selected the adherent cells (MSC) through a media change. The CS20BR08 was incubated in a humidified incubator at 37 °C with 7% CO_2_ for approximately 28 days using the CSBM-A06 (CORESTEM, Inc., Seoul, Korea) media containing 10% fetal bovine serum (Life Technologies Corporation, Grand Island, NY, USA), L-alanyl-L-glutamine (Biochrom AG, Berlin, Germany) and penicillin-streptomycin (Biochrom AG, Berlin, Germany). In the final process of the CS20BR08 production, cell surface markers CD29, CD44, CD73, CD90 and CD 105 (BD Pharmingen, Heidelberg, Germany) and negative markers CD34 and CD45 (BD Pharmingen, Heidelberg, Germany) were confirmed by flow-cytometry for the subsequent characterization of the MSCs. Under the qualification control protocol, all sterility tests including safety from bacteria, fungi, viruses and mycoplasma were confirmed. The final products, CS20BR08, were suspended in cerebrospinal fluid at 1 × 10^6^ cells/kg and were administered to patients through an intrathecal injection twice four weeks apart. The study was approved by the Institutional Review Board of Kyungpook National University Chilgok Hospital (2017-12-002).

### 2.2. Case Study

A 62-year-old male patient visited our clinic due to his progressive gait disturbance that started two years ago. He also presented non-motor symptoms such as constipation and sleep apnea. The patient had no history of previous neurological disorders and relevant family history. Upon a neurologic examination, the finger-to-nose and heel-to-shin tests revealed dysmetria in both limbs. He showed cerebellar speech and an ataxic gait. The tandem gait was also impaired. The score on the Korean version of the Scale for the Assessment and Rating of Ataxia (K-SARA) was 10. Brain magnetic resonance imaging (MRI) showed a pontocerebellar atrophy whereas the autonomic function test was normal. The patient was diagnosed with SAOA because of no definite autonomic dysfunctions. None of the medications prescribed had any significant effects and the symptoms gradually became worse. At the age of 64, the patient was treated with allogeneic bone marrow-derived MSC therapy via the intrathecal route twice every four weeks using his 36-year-old son as a donor.

**Figure 1:**
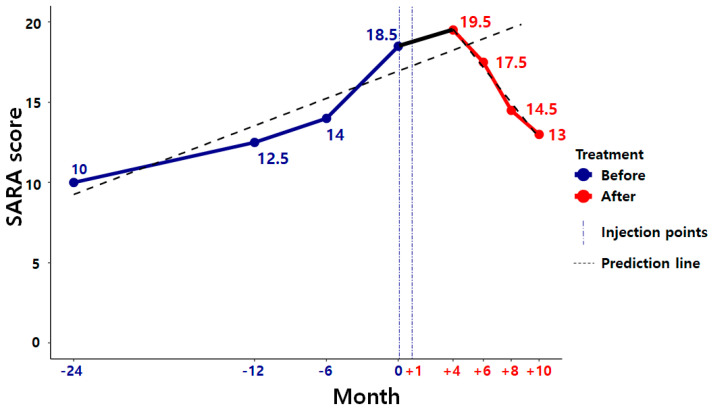
Line graph of the Korean version of the Scale for the Assessment and Rating of Ataxia (K-SARA) before and after the administration of the stem cell. K-SARA scores at the beginning of the stem cell administration had gradually deteriorated since the initial diagnosis (blue line) but showed a significant improvement up until 10 months (red line) following the completion of the second stem cell administration, contrary to the predicted line (black dot).

The K-SARA scores at the beginning of the stem cell administration had gradually deteriorated since the initial diagnosis. However, the subsequent scores revealed a gentle curve up to three months following the completion of the second stem cell administration. From then, the results demonstrated a significant improvement up to 10 months post-administration (Figure 1). The patient also reported a subjective improvement of motor symptoms. No adverse events were reported. Although follow-up was initially scheduled for a period of 12 months post-administration, it was only conducted for a period of 10 months due to COVID-19 restrictions.

## 3. Discussion

The degenerative nature of this disease dictates that the patient’s symptoms would gradually deteriorate. However, the patient’s symptoms confirmed a trend of steady improvement following 10 months of allogeneic MSC therapy [5]. An animal study showed that the administration of MSC therapy delayed the onset and ameliorated motor performances [6]. Two studies in humans have also shown that autologous MSC therapy via intra-arterial and intravenous [7] or intrathecal [8] routes could delay the progression of cerebellar dysfunction and improve functional status. Several mechanisms of MSC therapy for cerebellar ataxia have been proposed such as the mitigation of neuronal apoptosis, the regeneration of neurons and anti-inflammatory reactions [9,10]. In addition, a recent study has found that the growth rate and expression of the neuroprotective factors of MSCs in patients with cerebellar ataxia were decreased as opposed to MSCs in healthy people [10]. Therefore, it can be suggested that allogeneic MSC therapy via the intrathecal route in this patient might induce a neuroprotective effect, which, in our case, could be represented by the improved K-SARA scores.

## 4. Conclusions

Stem cell therapy may serve as a potential therapeutic option for degenerative cerebellar ataxia. However, it is important to evaluate and identify how long this effect will last as follow-up was discontinued after 10 months due to COVID-19 restrictions. In addition, the placebo effect could not be ruled out. Further studies will need to be carried out on large population samples to validate our hypothesis.

## Data Availability

All data and material supporting our findings are contained within the manuscript.
